# Spectral correction of light emitting diodes enables accurate hydration ratio calculation using narrowband diffuse reflectance spectroscopy

**DOI:** 10.1117/1.JBO.28.7.075005

**Published:** 2023-07-31

**Authors:** Jesse H. Lam, Jeonghun Kim, Kelsey J. Tu, Dongbin Kim, Sehwan Kim

**Affiliations:** a Dankook University, Beckman Laser Institute Korea, School of Medicine, Cheonan, Republic of Korea; b Dankook University, Department of Biomedical Engineering, Cheonan, Republic of Korea; c Dankook University, MEDiThings Co. Ltd., Industry-Academia Cooperation, Cheonan, Republic of Korea; d University of California, Irvine, Beckman Laser Institute, Department of Biomedical Engineering, Irvine, California, United States

**Keywords:** light emitting diode spectral correction, hydration, narrowband diffuse reflectance spectroscopy

## Abstract

**Significance:**

Light emitting diodes (LEDs) are commonly utilized for tissue spectroscopy due to their small size, low cost, and simplicity. However, LEDs are often approximated as single-wavelength devices despite having relatively broad spectral bandwidths. When paired with photodiodes, the wavelength information of detected light cannot be resolved. This can result in errors during chromophore concentration calculations. These errors are particularly apparent when analyzing water and fat in the 900 to 1000 nm window where the spectral bandwidth of LEDs can encompass much of the analysis region, resulting in intense crosstalk.

**Aim:**

We utilize and present a spectral correction (SC) algorithm to correct for the spectral bandwidth of LEDs. We show the efficacy using a narrowband technique of spectrally broad and overlapping LEDs.

**Approach:**

Narrowband diffuse reflectance spectroscopy (nb-DRS), a technique capable of quantifying the hydration ratio (${\mathrm{RH}}_{2}O$) of turbid media, was utilized. nb-DRS typically requires a broadband light source and spectrometer. We reduce the hardware to just five LEDs and a photodiode detector, relying on SC to compensate for spectral crosstalk. The effectiveness of our SC approach was tested in simulations as well as in an emulsion phantom and limited selection of human tissue.

**Results:**

In simulations, we show that calculated ${\mathrm{RH}}_{2}O$ errors increased with the spectral bandwidth of LEDs but could be corrected using SC. Likewise, in emulsions, we found an average error of 8.7% (maximum error 14%) if SC was not used. By contrast, applying SC reduced the average error to 2.2% (maximum error of 6.4%). We show that despite utilizing multiple, spectrally broad, and overlapping LEDs, SC was still able to restore the performance of our narrowband method, making it comparable to a much larger full broadband system.

## 1 Introduction

Maintaining hydration is necessary for human survival, yet it remains a challenge to monitor continuously and portably by optical methods. Dehydration can result in severe athletic and cognitive deterioration under 10% body water loss, and potential death if it further progresses.^1^^,^^2^ Tissue hydration was also found to be an important biomarker in other fields, such as breast cancer research,^3^^–^^5^ dermatology,^6^^,^^7^ and critical care medicine.^8^^,^^9^ Approaches for estimating water content have been previously validated using near-infrared (NIR) optical techniques, such as diffuse optical spectroscopy^10^^–^^12^ and spatial frequency domain imaging (SFDI).^13^^,^^14^

In our previous work, we demonstrated that narrowband diffuse reflectance spectroscopy (nb-DRS) can calculate the hydration ratio (${\mathrm{RH}}_{2}O$), which tracked closely with absolute water fraction. This was achieved by analysis of diffuse reflectance in the far NIR 900 to 1000 nm wavelength region^15^ using a broadband lamp and spectrometer. We defined ${\mathrm{RH}}_{2}O$ as a ratio of water ($H_{2}O$) and lipid (FAT):

$${\mathrm{RH}}_{2}O\;(\%)=100\times \frac{H_{2}O}{H_{2}O+\mathrm{FAT}}.$$ (1)

Due to the low hardware requirements of nb-DRS, we theorized that it could be a suitable candidate for miniaturization as a wearable hydration monitor. For the light source, multiple wavelength light emitting diodes (LEDs) could be utilized in lieu of the broadband lamp as a smaller, lower power, and low-cost substitute.^16^

However, for detection, transitioning from spectrometers to photodiodes has proved to be challenging. One drawback is the loss of high-resolution spectral data as photodiodes cannot resolve wavelength information. This limitation is exacerbated by LEDs having a non-negligible spectral full-width at half-maximum (FWHM), potentially leading to spectral crosstalk if not corrected.^17^^–^^19^ For some spectroscopic techniques, LEDs can be approximated using the peak wavelength. Using this approach, Mazhar et al.^20^ reported a <5% error for SFDI when the FWHM of LEDs were below 30 nm. Kurata et al.^21^^,^^22^ proposed averaging the chromophore extinction coefficients based on the bandwidth of the sources’ LEDs. However, this approach is not compatible with nb-DRS as a narrowband (900 to 1000 nm) technique. Utilizing the average extinction coefficients of given broad (>30  nm) LEDs is not feasible as the average would encompass too much of our analysis region including both $H_{2}O$ and FAT absorption peaks. Other groups have adopted an integration approach that directly uses the LED emission spectra during calculations.^23^^–^^26^ This method requires careful characterization of the system LEDs and is more appropriate for nb-DRS.

We utilize and present a specific integration algorithm for spectral correction (SC) that can mitigate crosstalk errors for narrowband techniques even when using multiple, spectrally broad, and overlapping LEDs. We show the effectiveness of this algorithm in simulations and an emulsion phantom spanning a wide range of ${\mathrm{RH}}_{2}O$ values. Measurements of human abdomen and thenar tissue are also performed. Pseudo-code and MATLAB code for our application of SC for nb-DRS are also provided. We hope that our efforts will facilitate the development of tissue hydration monitors using nb-DRS and encourage application of SC to other modalities as well.

## 2 Materials and Methods

### 2.1 Optical Instrumentation

We constructed an nb-DRS probe utilizing two approaches to the technique. To serve as a reference device, a full broadband nb-DRS method was used, as previously reported.^15^ A tungsten halogen lamp (HL-2000-HP, Ocean Insight) was coupled using an optical fiber with a core diameter of 400  μm. For detection, a spectrometer (ULS2048XL, Avantes B.V.), which was fiber coupled using a 1000  μm diameter optical fiber, was utilized.

**Fig. 1:**
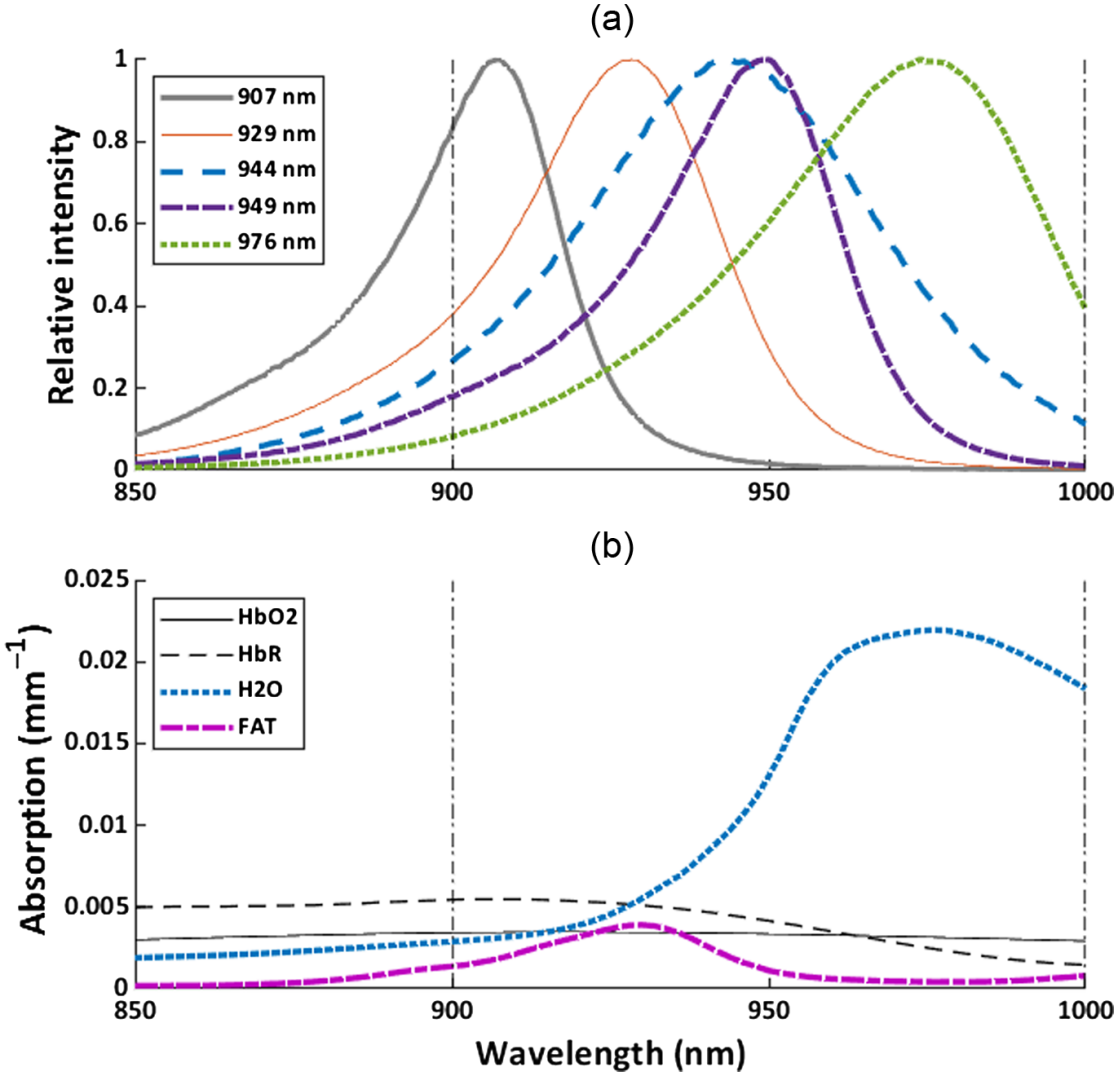
Spectra of the LEDs in the device. Vertical dashed-dot lines indicate the wavelength analysis region for nb-DRS. (a) Spectra for the LEDs in the nb-DRS instrument. LEDs are labeled by their peak wavelengths, shown in the legend. (b) Chromophore absorption spectra for human breast tissue containing 12.6  μM ${\mathrm{HbO}}_{2}$, 27.9  μM HbR, 45.1% $H_{2}O$, and 29.8% FAT.^27^^–^^29^ It can be seen that the spectral bandwidth of several LEDs were wide enough to overlap each other as well as both the $H_{2}O$ and FAT absorption peaks in this wavelength range.

As for the other nb-DRS approach, we utilized a customized multi-package LED containing five LEDs (Shenzhen Best LED Opto-electronic Co., Ltd.) with spectra shown in Fig. 1(a). The peak wavelengths of the LEDs were 907, 929, 944, 949, and 976 nm, whereas the FWHM were 29, 38, 56, 35, and 53 nm, respectively. Compared with typical absorption spectra for tissue containing oxyhemoglobin (${\mathrm{HbO}}_{2}$), deoxyhemoglobin (HbR), $H_{2}O$, and FAT chromophores, e.g., human breast tissue^27^ shown in Fig. 1(b), it can be seen that our LED spectra were quite broad. In particular, the 944, 949, and 976 nm LED spectra significantly overlapped with both the FAT and $H_{2}O$ absorption peaks. There was also significant overlap between LEDs. A photodiode (S121C, Thorlabs, Inc.), which was fiber coupled by a 1000  μm optical fiber, was used for detection. The LED and broadband approaches were mounted in a cross-configuration.

Optical components were mounted in a single 3D-printed probe with a source to detector distance of 15 mm. Due to the slight spatial distribution of the LEDs in the package, the actual source–detector separation for each individual LED varied between 13.5 and 15.5 mm, as measured using a caliper. A 0.6 mm thick transparent plastic sheet was placed between the probe and emulsion to protect the optical components. Possible effects of light piping due to the plastic sheet were empirically found to be negligible, with tissue ${\mathrm{RH}}_{2}O$ calculations only differing by ∼1%. Device calibration and LED spectra were measured using a reflectance standard (SRS-02-020, Labsphere Inc.). Using the measurement from the reflectance standard as a reference, instrument characteristics, such as different LED powers for each wavelength and photodiode sensitivity, can be approximated. However, we note that, without a direct measurement of the photodiode responsivity, the detector wavelength sensitivity function cannot be perfectly corrected and may contribute to experimental error.

The LEDs were powered and controlled using an Arduino Nano. The broadband lamp, spectrometer, and photodiode were operated using custom MATLAB 2021b (The MathWorks, Inc.) code.

### 2.2 Simulations

A set of simulated broadband reflectance spectra were generated using the steady-state diffusion model^30^ with varying ${\mathrm{RH}}_{2}O$ values and a constant reduced scattering of $1\;{\mathrm{mm}}^{-1}$. LED profiles with center wavelengths equal to the ones in our device were simulated as Gaussian curves with FWHM values increasing from 10 to 60 nm. The simulated broadband reflectance was then transformed into simulated LED reflectance using the Gaussian curves.

We then attempted to calculate ${\mathrm{RH}}_{2}O$ by two methods. First, we used the original nb-DRS algorithm, which does not account for the spectrum of the light source. Instead, the LEDs were assumed to be represented by the peak wavelength. Second, we used our SC algorithm, which assumes data from the photodiode to be an integration of reflectance over wavelength based on the measured LED spectra.

### 2.3 Sample Measurements

An emulsion phantom was constructed following processes similar to those described by Ohmae et al.,^11^ with slight modifications to maintain the phantom in liquid form instead of a solid gelatin. An oil and water emulsion phantom was fabricated with an initial oil to water ratio of 90 to 10 using soy lecithin (2% by weight) as the emulsifier. The solutions were then mixed using a high-speed vacuum blender (i8800, Jiaxiang Electric Co., Ltd.). Next, the phantom was diluted by adding 100 mL of water for each step. By maintaining water as the continuous medium, the phantom could be diluted without separation of the constituents.^31^ This approach also allowed for measurements across a large range of phantom ${\mathrm{RH}}_{2}O$ values without needing to construct a new phantom for each step. After 15 dilution steps, the final expected ${\mathrm{RH}}_{2}O$ was 74.3%. After every step, the phantom was homogenized using a hand-held mixer. The probe was also lifted, cleaned, and then replaced such that the face of the probe rested on the liquid surface and was not submerged.

Finally, measurements were performed on two healthy human subjects (31 year old male and 30 year old female). Abdomen and thenar sites were selected as representative examples of fatty and lean tissue, respectively. The source–detector separation for the LED and photodiode combination was shortened to 11 mm to improve the signal-to-noise ratio. The source–detector separation for the broadband nb-DRS optical fibers remained unchanged. Although not a significant factor for homogeneous samples, such as emulsions, the differences in source–detector separation and optical geometry could result in slightly different measurement volumes in tissue. Hemoglobin was included during chromophore fits.

### 2.4 Data Processing

Data processing was done by custom code written in MATLAB. Our SC algorithm was employed to account for the spectral bandwidth of our LEDs, with the pseudo-code shown in Table 1. First, calibration was performed using a reflectance standard. In step 2, the measured LED profile data were normalized with respect to the maximum intensity for each wavelength to be used as weighting in a later step. Next, broadband total absorption was calculated using known chromophore extinction coefficient tables and an initial chromophore concentration guess (e.g., 50% $H_{2}O$ and 50% FAT). Similar to our previous work, we assumed a flat, constant scattering of $1.0\;{\mathrm{mm}}^{-1}$. Due to the power law of scattering, this was found to be a reasonable assumption in the far NIR of 900 to 1000 nm. Furthermore, as shown in our previous work, this scattering assumption has little effect on calculated ${\mathrm{RH}}_{2}O$.^15^ Broadband reflectance was then calculated using absorption and reduced scattering values. The high-spectral resolution broadband reflectance data were transformed into low-resolution simulated photodiode reflectance (e.g., 1 data point per LED) by multiplication with the LED profile weights and then summed over wavelength. The simulated photodiode reflectance was then minimized by comparison with the actual measured data with ${\mathrm{RH}}_{2}O$ as the final output.

**Table 1:** Pseudo-code detailing SC used for our nb-DRS system.

Pseudo-code: SC algorithm for nb-DRS
INPUT:
D are measurement data from the photodiode for each LED
C are calibration data for the photodiode for each LED
S are the LED spectral measurements
ε are the chromophore extinction coefficients for all wavelengths
λ are the wavelengths for extinction coefficients
N are the chromophore concentrations
f is the steady-state diffusion model function
θ are all other diffusion model inputs (e.g., index of refraction and scattering)
OUTPUT:
H is the calculated hydration ratio
START:
1. CALIBRATE measurement data D using calibration data C
$R=\frac{D}{C}$
2. NORMALIZE spectral information of the LEDs S
$S_{\text{norm}}=\frac{S}{\max(S)}$
3. FIT to solve for chromophore concentration N
$N=\arg\;\underset{N}{\min}{\left|R-R_{\text{norm}}(N;\theta )\right|}^{2}$
WHERE:
FOR j=1 to length of R
$R_{\text{sim}}(j)=\underset{\lambda }{\sum }[f(N,\varepsilon ,\lambda ,\theta )\times S_{\text{norm}}(\lambda ,j)]$
END
$R_{\text{norm}}=R(1)\times \frac{R_{\mathrm{si}}}{R_{\text{sim}}(1)}$
4. RETURN final output H
$H=100\times \frac{N_{H_{2}O}}{N_{H_{2}O}+N_{\mathrm{FAT}}}$

## 3 Results and Discussion

### 3.1 Simulations

**Fig. 2:**
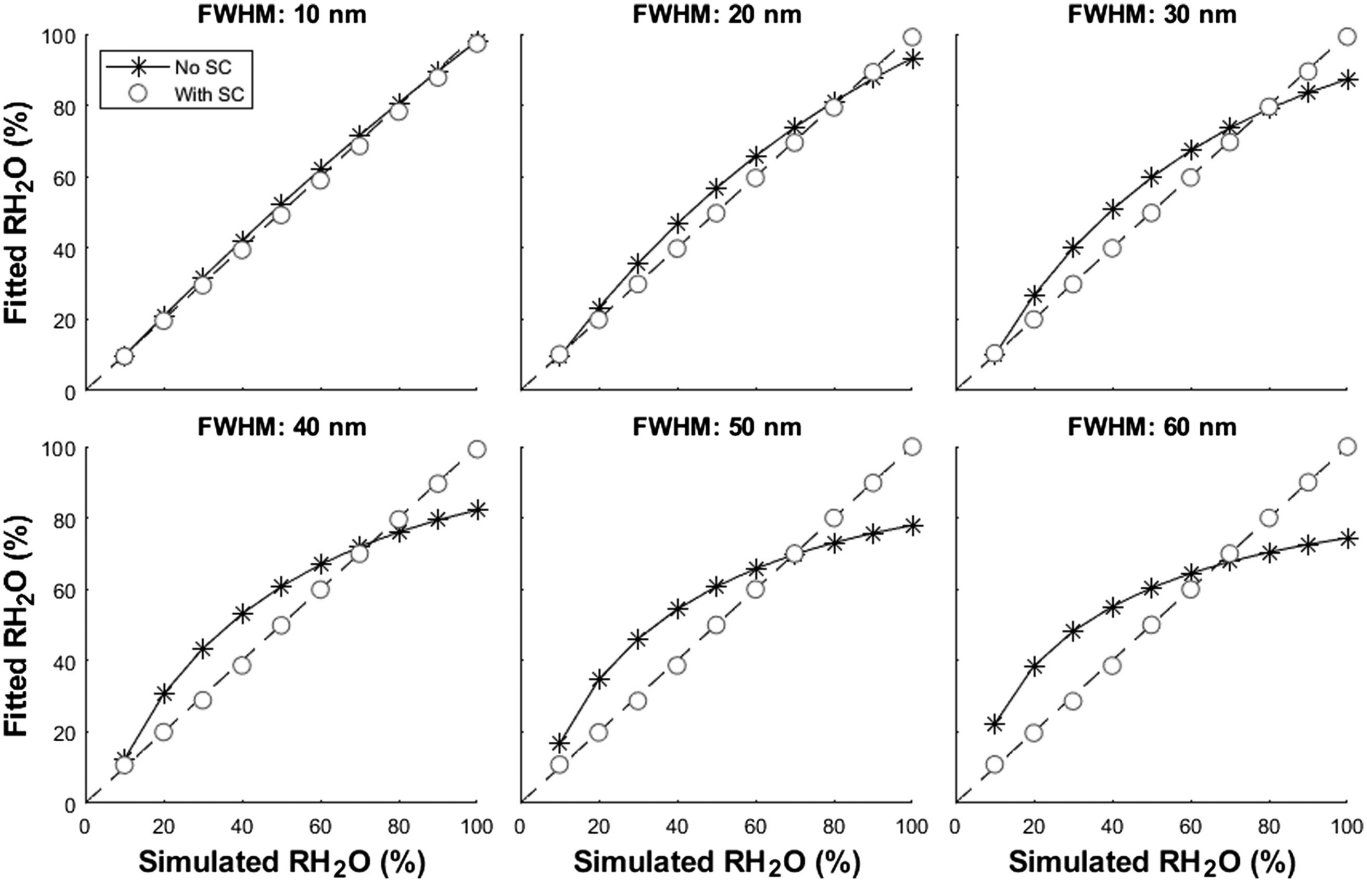
Simulated data to compare fitting approaches with and without our SC algorithm. The dashed line represents the line of identity. In the absence of SC, light from each LED was assumed to be the peak wavelength. When the FWHM of the LEDs was sufficiently narrow, this assumption was reasonable. However, as the spectral width of the LED increased, this assumption became invalid and the estimated ${\mathrm{RH}}_{2}O$ was increasingly erroneous. By contrast, using our SC algorithm, an accurate ${\mathrm{RH}}_{2}O$ was recovered despite increasing FWHM values.

A set of reflectance spectra were simulated using LEDs with increasing FWHM ranging from 10 to 60 nm. ${\mathrm{RH}}_{2}O$ values were then recovered using nb-DRS with and without our SC algorithm, as shown in Fig. 2. When using LEDs with an FWHM of only 10 nm, the performance for either approach was similar (∼1% error). However, as the bandwidth of the LEDs increased, the calculations without SC became progressively erroneous. As the FWHM reached 60 nm, the average absolute error had ballooned to 13.4%. By contrast, calculations of ${\mathrm{RH}}_{2}O$ with our SC algorithm maintained the same level of accuracy for all simulated FWHM values (within ∼1%).

### 3.2 Emulsion Phantoms

**Fig. 3:**
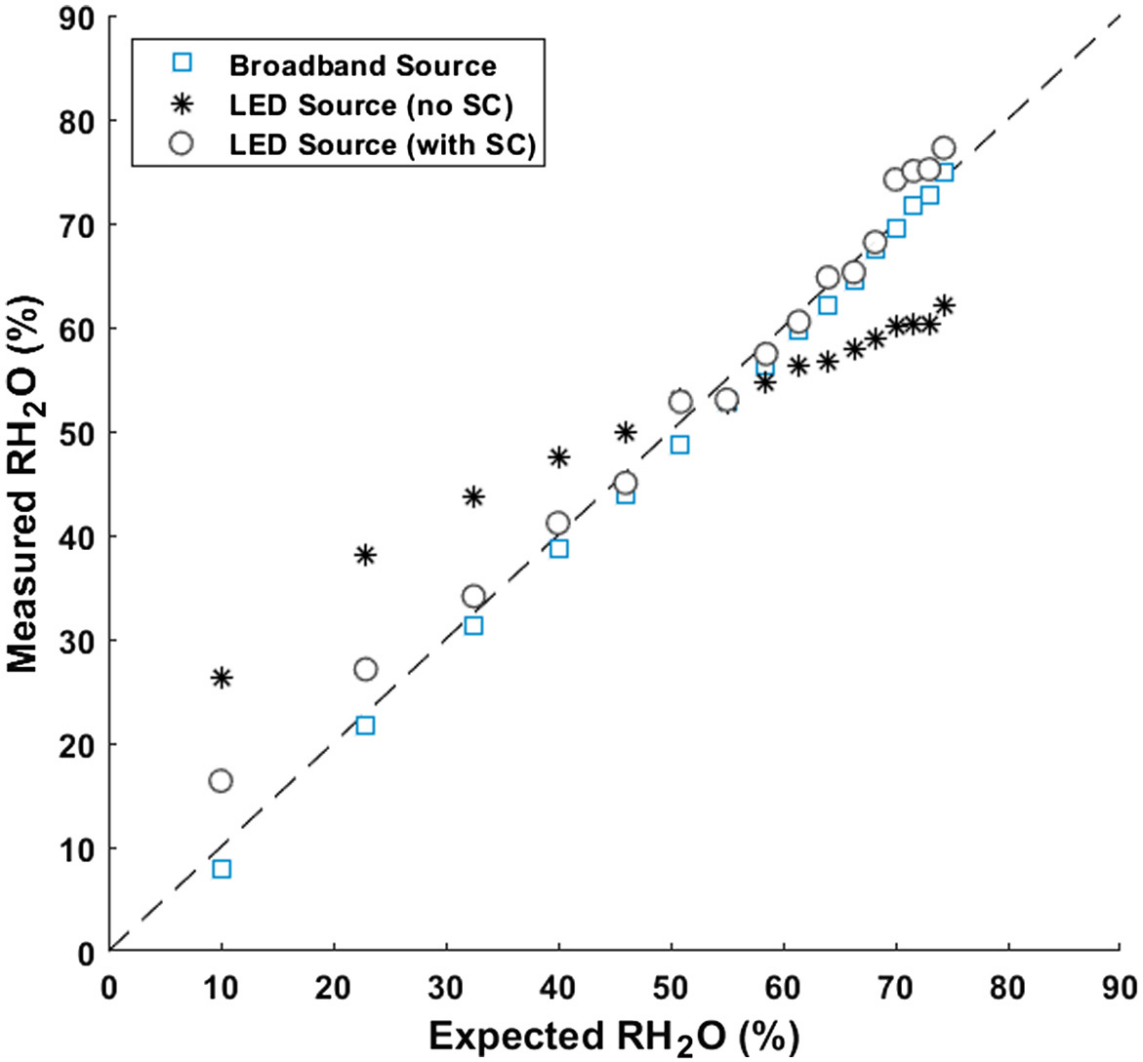
Emulsion ${\mathrm{RH}}_{2}O$ measured by nb-DRS using a broadband light source, LED source without SC, and LED source with SC. The dashed line represents the line of identity. Note that the dilution volume added at each step was maintained at 100 mL, whereas the total phantom volume increased over time. This resulted in a non-linear dilution trend, with larger ${\mathrm{RH}}_{2}O$ increases at the start of the experiment and smaller ${\mathrm{RH}}_{2}O$ changes near the end.

Our SC algorithm was further tested using an emulsion phantom. Three methods were compared, as seen in Fig. 3. First, the reference system, which used a broadband light source and spectrometer detector, is shown. This method performed most accurately with an average and standard deviation error of 1.4%±0.7%. The maximum error was 2.2%.

Next, the LEDs were utilized in conjunction with a photodiode detector. Effects of spectral crosstalk can be recognized not only by accuracy but also by sensitivity. For example, an LED at 929 nm might be considered sensitive to FAT, as the peak wavelength is matched with the lipid absorption peak. However, in reality, both $H_{2}O$ and FAT can affect reflectance measurements using this LED due to the wide spectral bandwidth. This was evident as, without SC, the dynamic range was poor. This was observed in both our simulations and emulsion phantom experiments. The error on the emulsion was found to be 8.7%±4.4% with a maximum error of 14.1%. Finally, using the same dataset, our SC algorithm was applied. This improved the error to 2.2%±1.7% with a maximum error of 6.4%. It can also be seen that applying SC restored the sensitivity, with a slope comparable to the full broadband approach.

Some discrepancy can be seen between the broadband approach and the LED (with SC) approach. The broadband approach has a much greater spectral resolution with 51 data points, allowing for more robust chromophore fitting. By contrast, the LED approach only has one datapoint for each LED. Furthermore, the LEDs are light sources with spectral bandwidths ranging from 30 to 50 nm. This spectral crosstalk can be corrected using our algorithm, but this lack of information cannot be perfectly compensated. Thus there are some small discrepancies between the broadband and LED (with SC) ${\mathrm{RH}}_{2}O$ results.

In addition, attenuation by scattering was most intense at the beginning of the experiment when the lipid droplet concentration was highest. Although the photodiode in use had a large active diameter of 9.7 mm, it was fiber-coupled to a 1 mm core diameter optical fiber to produce a more compact probe and allow for a shorter source-detector separation. Future iterations of this device will include custom circuitry with a direct-contact photodiode to improve the signal-to-noise ratio of our measurements.

### 3.3 Tissue Measurements

Measurement results on human tissue are shown in Table 2. The broadband and LED (with SC) approaches were able to clearly differentiate between abdomen and thenar tissue for the two subjects. ${\mathrm{RH}}_{2}O$ on abdomen tissue was low due to the significant presence of adipose, ranging from 18.9% to 37.8% for the broadband approach and 22.7% to 37.8% for the LED (with SC) approach. By contrast, thenar tissue was a much leaner tissue with ∼77% as reported by the broadband system. Using SC, the LED system reported a range between 69% and 80.7%. This discrepancy could be due to slightly different volume measurements due to the differences in source–detector separation (15 versus 11 mm). For either approach, the two tissue types were clearly delineated, with abdomen tissue having a much lower ${\mathrm{RH}}_{2}O$ than thenar, as expected. The last approach, LED (no SC), failed to properly fit the data. Although the emulsion only contained $H_{2}O$ and FAT, the inclusion of hemoglobin factors in live tissue likely exacerbated spectral crosstalk errors. Although SC can improve the performance of the LED approach, the technique is still ultimately limited by the number of LED and lack of high-resolution spectral information. Complex tissue is more computationally challenging due to the presence of other factors, such as hemoglobin, as well as other unaccounted chromophores. Accuracy could potentially be improved by the addition of LEDs and inclusion of other chromophores, such as myoglobin and melanin.

**Table 2:** ${\mathrm{RH}}_{2}O$ estimations on human abdomen and thenar tissue. The broadband device utilized a 15 mm source–detector distance, whereas the LED device utilized an 11 mm source–detector distance.

Tissue	Broadband (%)	LED (with SC) (%)	LED (no SC) (%)
Abdomen 1	37.8	37.8	58.2
Abdomen 2	18.9	22.7	53.6
Thenar 1	75.0	80.7	60.6
Thenar 2	78.7	69.0	59.7

## 4 Conclusion

We reported an SC algorithm that was shown to increase the accuracy of ${\mathrm{RH}}_{2}O$ measurements using nb-DRS despite the usage of overlapping, broad spectrum LEDs in a narrow wavelength region. This was achieved by leveraging the known spectra of the LEDs during chromophore calculations. We showed the efficacy of SC in simulations as well as an emulsion phantom and a limited selection of tissues using nb-DRS. Through this work, we hope to facilitate the simplification and miniaturization of spectroscopic technologies and encourage the usage of SC, especially for narrowband LED-based devices.

## Code, Data, and Materials Availability

Application of the algorithm for nb-DRS is available for viewing at Code Ocean (https://doi.org/10.24433/CO.7379307.v1). Referenced chromophore extinction coefficient tables were not republished here but can be found in the original cited sources.
